# Proteomic Studies for the Identification and Characterization of Marine Bioactive Molecules

**DOI:** 10.3390/md23110433

**Published:** 2025-11-10

**Authors:** Nedeljka Rosic

**Affiliations:** 1 Faculty of Health, Southern Cross University, Gold Coast, QLD 4225, Australia; nedeljka.rosic@scu.edu.au; 2 Marine Ecology Research Centre, Southern Cross University, Lismore, NSW 2480, Australia

The marine environment is a rich source of natural products that, as promising bioactive compounds, demonstrate environmentally friendly potential for application across various industries [1,2]. These bioactive molecules may be used in environmental protection, supporting the transition to green sectors, and promoting the sustainable use of natural resources [3,4,5,6]. Proteomic studies play a crucial role in this process by analyzing the activity, stability, degradation, modification, and interactions of proteins, as well as their influence on metabolic pathways [7,8,9]. This approach also assesses variations in protein expression levels and the impact of differential gene expression on proteomic profiles [10,11]. Advances in proteomics, particularly through the integration of cutting-edge technologies such as mass spectrometry-based protein interactome studies, artificial intelligence (AI)-based approaches, and machine learning techniques are opening new frontiers in modern-day research explorations that were not possible in the past [12,13,14]. This rapid progress is especially vital in the medical field, where there is an urgent demand for novel and more effective therapeutic agents, disease biomarkers and addressing the needs of the aging population [15,16]. Moreover, proteomic insights are deepening our understanding of the bioactive potential of marine-derived bioproducts, paving the way for innovative applications in health and biotechnology.

Over the last few decades, proteomics research has tremendously expanded, including advancements via high-throughput techniques resulting in faster and more reliable proteome profiles, a better understanding of biological mechanisms, and the discovery of new natural products [6,17,18]. The use of proteomics to facilitate the application of algal natural products in commercial purposes requires the effective use of specific proteomics methodologies combining assessments of bioactivity analyses, such as anti-bacterial, anti-proliferation, anti-viral, anti-aging, anti-fungal, and anti-inflammatory activities [19,20,21,22]. This Special Issue, entitled “Proteomic Studies for the Identification and Characterization of Marine Bioactive Molecules” (https://www.mdpi.com/journal/marinedrugs/special_issues/747IJQ0OF9, accessed on 24 October 2025) focuses on the application of proteomics in the utilization of marine species as a source of valuable pharmacologically active compounds. As a result of growing interest in proteomics research, this Special Issue has gathered ten original articles and reviews.

This editorial aims to provide an overview of recent advances in proteomics and its application for the discovery and characterization of marine bioactive molecules, as published in this Special Issue “Proteomic Studies for the Identification and Characterization of Marine Bioactive Molecules”. The contributions from this issue are listed below within the List of Contributions.

*Contribution 1:* https://doi.org/10.3390/md23030102

Sun et al. analyzed protein components using sodium dodecyl sulfate–polyacrylamide gel electrophoresis (SDS–PAGE) on crude venoms from five vermivorous cone snails and compared their composition and bioactivity. In addition, reverse-phase HPLC (RP-HPLC) fingerprinting and mitochondrial cytochrome c oxidase I (COI) gene clustering revealed that venom diversity was genetically correlated among species. All venoms were lethal to insects and zebrafish, with *Conus quercinus* showing the strongest insecticidal activity (LD_50_ = 0.6 μg/mg) and *C. tessellatus* exhibiting the highest zebrafish lethality (LD_50_ = 0.2 μg/mg). Four species’ venoms displayed cytotoxicity against ovarian cancer cells, while only *C. caracteristicus* showed significant analgesic effects. These findings identify several cone snail species with promising bioactivities, underscoring their potential for developing novel marine-derived peptide drugs and highlighting the importance of exploring the South China Sea’s biodiversity for pharmaceutical discovery.

*Contribution 2:* https://doi.org/10.3390/md23020082

Cunha et al. used high-performance size-exclusion chromatography to explore bioactive peptides from fish body mucus of *Halobatrachus didactylus*, a species inhabiting pollutant-exposed intertidal zones. In silico computational assessment of peptide aggregation propensity was used to further assess the aggregation propensities of selected peptides. In vitro studies revealed peptide aggregation at specific ionic strengths (344 and 700 mM) and neutral pH. Though none of the analyzed peptides showed antimicrobial activity, they inhibited *Pseudomonas aeruginosa* biofilm formation. HdVLPN and HdLPN peptides showed strong antioxidant activity, decreasing at acidic pH, while HdPPP peptide displayed antihypertensive and antidiabetic potential through ACE and α-glucosidase inhibition. The study highlights the importance of validating peptide bioactivities in their native mucus environment and conserving this species as a potential source of novel biotechnological compounds.

*Contribution 3:* https://doi.org/10.3390/md22100470

Hua et al. explored the South China Sea’s sea anemone resources, including *Macrodactyla doreensis*, containing toxins rich in proteins and peptides with potential biotechnological value. This study conducted comprehensive omics analyses of different tissues (i.e., the tentacles and column), including transcriptomic and proteomics in silico modelling to construct putative protein and peptide databases and compare their profiles utilizing multiple bioinformatics tools (e.g., the Pfam database, Multiple sequence alignment MUSCLE, AlphaFold2 Modeling). Among 4239 identified transcripts, putative proteins accounted for 81.53%, mainly immunoglobulins and proteases concentrated in the column, linked to biological functions. Putative peptides (18.47%) were primarily found in the tentacles, featuring ShK-domain and Kunitz-type peptides associated with predatory behavior. Forty peptides across eight typical families were identified, and their 3D structures and potential targets were predicted using AlphaFold2 and molecular docking. The findings highlight the molecular diversity and functional complexity of *M. doreensis* toxins, demonstrating an effective approach for predicting peptide structure and activity, supporting future discovery of bioactive compounds from marine organisms.

*Contribution 4:* https://doi.org/10.3390/md22100468

Tassara et al. employed proteomic and biochemical methods to analyze the crude nematocyst extract of *Velella velella*, a common hydrozoan harmless to humans. The analysis identified 783 proteins, including structural components, enzymes, and potential toxins, revealing a venom composition similar to more toxic cnidarians. Biochemical assays confirmed active hydrolytic enzymes such as proteases, phospholipases, hyaluronidases, DNases, and chitinases within the extract. Proteomics allowed the detection of trace compounds with minimal animal collection, important for ecosystem preservation. These findings provide a valuable resource for future research exploring the pharmacological applications of *V. velella* venom components through recombinant production and functional studies, advancing the development of novel marine-derived biotechnological tools.

*Contribution 5:* https://doi.org/10.3390/md22030113

Kureara et al. investigated the protective effects of oyster (*Crassostrea nippona*) extracts against dexamethasone-induced muscle atrophy in C2C12 cells. Extracts were enzymatically hydrolyzed using alcalase (AOH), flavourzyme (FOH), and protamex (POH). Among them, AOH showed the strongest effects, enhancing cell proliferation and promoting dose-dependent myotube formation. The conventional polyacrylamide gel-based method, SDS-PAGE, was used to evaluate the molecular weight and amino acid composition of AOH. Western blot and RT-qPCR analyses revealed that AOH downregulated atrophy-related proteins (MuRF-1, Atrogin, Smad2/3, and Foxo-3a) while upregulating myogenesis-related markers (myogenin, MyoD, myosin heavy chain, and mTOR). These findings indicate that AOH modulates the ubiquitin–proteasome and mTOR signaling pathways to mitigate muscle degradation and promote muscle growth. Overall, *C. nippona* hydrolysate, particularly AOH, shows promise as a bioactive ingredient for developing functional foods aimed at preventing or alleviating muscle atrophy and related conditions such as sarcopenia.

*Contribution 6:* https://doi.org/10.3390/md22020071

Li et al. targeted sea anemone venoms, which contain diverse toxins that may aid in both prey capture and predator defense. This study applied integrated transcriptomic and proteomic “venomics” to characterize the toxins of *Heteractis magnifica*. Transcriptomic analysis identified 728 putative toxin sequences (442 from tentacles and 381 from the column), which were classified into 68 gene superfamilies. Proteomic analysis confirmed 101 proteins and peptides (91 detected in tentacles and 39 in the column). Several toxin types, including ShK-like peptides and defensins, were co-expressed in both tissues. Homology-based modeling further predicted the 3D structures and potential functions of seven representative toxins. Collectively, this comprehensive venomics approach revealed the molecular diversity and tissue distribution of *H. magnifica* toxins, enhancing understanding of cnidarian venom systems and providing a valuable foundation for future marine drug discovery and development.

*Contribution 7:* https://doi.org/10.3390/md22010012

Ramirez-Carreto et al. explored sea anemones as valuable sources of bioactive polypeptides by performing a proteomic analysis of crude venom extracts from *Anthopleura dowii* and *Lebrunia neglecta*, identifying 201 polypeptides, 39% of which were common to both species. The identified proteins included hydrolases, oxidoreductases, transferases, heat shock proteins, adhesion proteins, and protease inhibitors. Functional annotation and interaction analyses revealed that these proteins are mainly involved in endoplasmic reticulum-related metabolic processes, such as carbon metabolism and protein processing. Additionally, several oxidative-stress-related enzymes, including superoxide dismutase, peroxiredoxins, thioredoxin, and glutathione oxidase, were detected. Overall, the findings provide new insights into the complex polypeptide composition of sea anemone venoms and highlight their potential as a source of novel bioactive molecules for developing therapeutic agents and biotechnological applications.

*Contribution 8:* https://doi.org/10.3390/md21020111

Jiang et al. assessed *Tachypleus tridentatus* (horseshoe crab), a marine organism used in traditional Chinese medicine, with its plasma known for various health benefits. This study investigated the effects of *T. tridentatus* plasma on bone growth in rats. Plasma components were separated by ultrafiltration and identified using mass spectrometry; samples with different molecular weight ranges were then tested. The 10–30 kDa plasma fraction showed the strongest bone-growth-promoting activity, outperforming whole plasma. Transcriptomic, proteomic, and other bioinformatics analyses revealed that peptides in this fraction, particularly those rich in specific amino acid residues, were associated with enhanced growth. Differentially expressed proteins were primarily enriched in the PI3K-AKT signaling pathway, a key regulator of cell proliferation and bone development. These findings suggest that *T. tridentatus* plasma, especially the 10–30 kDa fraction, holds promise as a bioactive growth-promoting agent with potential clinical applications in bone repair and regenerative medicine.

*Contribution 9:* https://doi.org/10.3390/md23040165

This review investigated the increasing demand for sustainable natural products in food, supplements, and cosmetics to slow aging and promote longevity. Both macro- and microalgae are rich sources of proteins, amino acids, fatty acids, vitamins, and minerals with potential health benefits. The biotechnological industry increasingly uses high-throughput omics technologies, especially proteomics, to identify anti-aging compounds from these natural resources. Proteomics enables detailed analysis of protein content, structure, function, and interactions, aiding understanding of molecular aging processes. Mass spectrometry-based and chemical proteomics techniques help identify and quantify proteins and natural products involved in aging regulation. This review highlights marine algae’s anti-aging compounds and novel proteomics methods, supporting their application in innovative and sustainable biotechnological solutions for healthy aging.

*Contribution 10:* https://doi.org/10.3390/md21120633

Rosic and Thornber explored current evidence on the growing patterns of marine macroalgae, or seaweeds, facing varying abiotic (light, temperature, nutrients, salinity) and biotic (grazing, pathogens) stresses, and their adaptive strategies, such as synthesis of secondary metabolites like Mycosporine-Like Amino Acids (MAAs). MAAs, as small, water-soluble compounds with UV-absorbing properties, have been noted for their antioxidative, anti-inflammatory, and photoprotective properties, which are valuable for biotechnological applications. However, their broader use is limited by low bioavailability, challenges in heterologous production, and scarcity in natural sources. Bloom-forming macroalgae from Chlorophyta, Phaeophyceae, and Rhodophyta clades occasionally produce massive biomass during blooms, offering a rich source of pharmacologically active compounds, including MAAs. This review highlights these bloom-forming species and explores how proteomics can advance their utilization, addressing current environmental and biotechnological hurdles to harness their full potential for sustainable applications.

In summary, this Editorial’s concluding remarks include gratitude to the Editorial Assistant, Managing Editors, and Editorial Board. Furthermore, the authors who have contributed their findings to this Special Issue are highly appreciated for their submissions and follow-up modifications that have increased knowledge and scientific understanding in the areas of proteomics and marine products. Lastly, we appreciate the help of the reviewers who thoroughly examined all the submitted manuscripts, allowing further progress in the field of proteomics and marine active compounds.

## References

[1] Carroll A.R., Copp B.R., Grkovic T., Keyzers R.A., Prinsep M.R. (2025). Marine natural products. Nat. Prod. Rep..

[2] Rosic N.N. (2021). Recent advances in the discovery of novel marine natural products and mycosporine-like amino acid UV-absorbing compounds. Appl. Microbiol. Biotechnol..

[3] Lauritano C., Ferrante M.I., Rogato A. (2019). Marine natural products from microalgae: An -omics overview. Mar. Drugs.

[4] Kumar M.S., Adki K.M. (2018). Marine natural products for multi-targeted cancer treatment: A future insight. Biomed. Pharmacother..

[5] Desriac F., Jégou C., Balnois E., Brillet B., Le Chevalier P., Fleury Y. (2013). Antimicrobial peptides from marine proteobacteria. Mar. Drugs.

[6] Ambrosino L., Tangherlini M., Colantuono C., Sangiovanni A.M., Miralto M., Sansone C., Chiusano M.L. (2019). Bioinformatics for Marine Products: An Overview of Resources, Bottlenecks, and Perspectives. Mar. Drugs.

[7] Zhang Y., Fonslow B.R., Shan B., Baek M.-C., Yates J.R. (2013). Protein Analysis by Shotgun/Bottom-up Proteomics. Chem. Rev..

[8] Graves P.R., Haystead T.A. (2002). Molecular biologist’s guide to proteomics. Microbiol. Mol. Biol. Rev..

[9] Al-Amrani S., Al-Jabri Z., Al-Zaabi A., Alshekaili J., Al-Khabori M. (2021). Proteomics: Concepts and applications in human medicine. World J. Biol. Chem..

[10] Ryu J., Park S.-J., Kim I.-H., Choi Y.H., Nam T.-J. (2014). Protective effect of porphyra-334 on UVA-induced photoaging in human skin fibroblasts. Int. J. Mol. Med..

[11] Rong D., Su Y., Jia D., Zeng Z., Yang Y., Wei D., Lu H., Cao Y. (2024). Experimentally validated oxidative stress -associated prognostic signatures describe the immune landscape and predict the drug response and prognosis of SKCM. Front. Immunol..

[12] Pashaei E., Liu S., Li K., Zang Y., Yang L., Lautenschlaeger T., Huang J., Lu X., Wan J. (2025). DiCE: Differential centrality-ensemble analysis based on gene expression profiles and protein–protein interaction network. Nucleic Acids Res..

[13] Tan C., Zhang Z., Liu H., Liu X., Ai Y., Wu X., Jian E., Song Y., Yang J. (2025). Progress and trends on machine learning in proteomics during 1997-2024: A bibliometric analysis. Front. Med..

[14] Wu S., Zhang S., Liu C.-M., Fernie A.R., Yan S. (2025). Recent Advances in Mass Spectrometry-Based Protein Interactome Studies. Mol. Cell. Proteom..

[15] Argentieri M.A., Xiao S., Bennett D., Winchester L., Nevado-Holgado A.J., Ghose U., Albukhari A., Yao P., Mazidi M., Lv J. (2024). Proteomic aging clock predicts mortality and risk of common age-related diseases in diverse populations. Nat. Med..

[16] Gafken P.R., Paczesny S. (2025). Blood proteomics for quantitative biomarkers of cellular therapies. Biomark. Res..

[17] Wink M. (2008). Bioprospecting: The Search for Bioactive Lead Structures from Nature. Medicinal Plant Biotechnology: From Basic Research to Industrial Applications.

[18] Jagadeesh D.S., Kannegundla U., Reddy R.K. (2017). Application of proteomic tools in food quality and safety. Adv. Anim. Vet. Sci..

[19] Rosic N.N., Huang W., Johnston W.A., DeVoss J.J., Gillam E.M. (2007). Extending the diversity of cytochrome P450 enzymes by DNA family shuffling. Gene.

[20] Rosic N.N. (2019). Mycosporine-Like Amino Acids: Making the Foundation for Organic Personalised Sunscreens. Mar. Drugs.

[21] Rocha-Martin J., Harrington C., Dobson A.D., O’Gara F. (2014). Emerging strategies and integrated systems microbiology technologies for biodiscovery of marine bioactive compounds. Mar. Drugs.

[22] Bedoux G., Hardouin K., Burlot A.S., Bourgougnon N., Bourgougnon N. (2014). Chapter Twelve—Bioactive Components from Seaweeds: Cosmetic Applications and Future Development. Advances in Botanical Research.

